# Comment on Di Giosaffatte et al. A Novel Hypothesis on Choroideremia-Manifesting Female Carriers: Could *CHM* In-Frame Variants Exert a Dominant Negative Effect? A Case Report. *Genes* 2022, *13*, 1268

**DOI:** 10.3390/genes14122160

**Published:** 2023-11-29

**Authors:** Lewis E. Fry, Robert E. MacLaren

**Affiliations:** 1 Nuffield Department of Clinical Neuroscience, University of Oxford, Oxford OX3 9DU, UK; 2 The Royal Victorian Eye and Ear Hospital, East Melbourne, VIC 3002, Australia; 3 Oxford Eye Hospital, Oxford University Hospitals NHS Foundation Trust, Oxford OX3 9DU, UK

The recent publication of Di Giosaffatte et al. [1] challenges the conventional understanding of the variable phenotype observed in female carriers of mutations in *CHM*, the causative gene of choroideremia in affected males. Photoreceptors and RPE degenerate due to a prenylation deficiency caused by a lack of functional REP1 protein due to pathological *CHM* variants.

X-inactivation randomly silences either the paternal or maternal X-chromosome early in embryogenesis in each cell to ensure gene dosage compensation between male (XY) and female cells (XX) [2]. Clonal retinal progenitor populations that arise from cells with one inactivated X chromosome are heterogeneously distributed [3] in patterns that reflect the patchy degeneration seen in female carriers of *CHM.* Skewing of X-inactivation, where one X-chromosome is preferentially inactivated, is the most likely explanation for the variability of the severity of phenotype in female carriers [4]. The rates of either minimal or severe carrier phenotype is consistent with rates of skewed X-inactivation, estimated at around 8–10% of individuals with skewing > 80% [5].

Di Giosaffatte et al. highlight the escape of X-inactivation of *CHM* as an alternative mechanism for the carrier phenotype observed in their case of a family with a mutation disrupting the canonical splice site in *CHM* (c.1349+1G>A) causing in-frame skipping of exon 10. Their data lack assays to assess protein expression or in vitro prenylation function; however, in silico data predict a REP1 protein with a deleted region that might impair binding of Rab proteins and may sequester Rab Geranylgeranyl Transferase (RGGT), hence theoretically contributing to a dominant negative effect.

Escape genes are genes that can be expressed from the inactivated X-chromosome and are generally found at the tips of the X-chromosome. It has not been conclusively determined that *CHM* is either an escape gene or a variable escape gene (a gene that is variably expressed in some individuals or tissues). In Balaton’s 2015 consensus review, the escape status of *CHM* was indeterminant [6]. A model-based approach analysing bulk RNA-seq data suggested inter-individual heterogeneity, with up to 45% of females showing *CHM* expression escape [7]. Finally, Tukiainen et al. demonstrated sex-biased expression of *CHM* supported by allele-specific expression analysis of single-cell RNAseq data showing low-level escape in one of two cell lines analysed [8]. These studies have not clearly shown how they have differentiated expression of *CHM* from *CHML,* a highly homologous retrogene expressed from chromosome 1. Notably, two other escape genes (RBMX2 and RPL36A) identified by Tukiainen et al. also have retrogenes that may confound the measurement of their expression. As Di Giosaffatte et al. highlight, further work to investigate expression escape specifically in retinal tissue is needed.

If *CHM* does variably escape X-inactivation, it would then be expected that we would observe a significant number of phenotypically normal women due to the expression of a normal allele in affected cells. However as we and others have shown [4], while female carriers are often asymptomatic, careful examination and imaging invariably reveal a phenotype in almost all patients. Finally, imaging of the affected male (II:1, age 36) shows a large residual autofluorescence island, which is consistent with a mild phenotype of relatively slow degeneration for this patient’s age [9]. If the mechanism of the *CHM* (c.1349+1G>A) variant was dominant negative, a more severe phenotype would be expected in the affected male, consistent with the more severe female carrier phenotype. Moreover, the mild appearance would more likely be indicative that the deleted REP1 protein has a beneficial effect compared to a true null mutation [9].

The hypothesis presented by Di Giosaffatte et al. depends on the presence of four conditions: (1) that REP1 predominantly binds RGGT prior to Rab proteins according to the ‘alternative model’, (2) that the truncated protein sequesters RGGT and impairs Rab prenylation in vivo as predicted by in silico domain analysis in order to cause a dominant negative effect, (3) that escape expression of *CHM* occurs at sufficient levels for this to be pathogenic to cause a carrier phenotype and (4) that this mechanism is concordantly pathogenic in males to cause choroideremia. At present, there remains insufficient evidence to support these four conditions. Skewed X-inactivation continues to seem the more likely mechanism for the observed female phenotype; however, as this article highlights, further research into the possible escape expression of *CHM* from the inactivated X-chromosome in retinal tissue is warranted.
